# mTOR regulates proteasomal degradation and Dp1/E2F1- mediated transcription of KPNA2 in lung cancer cells

**DOI:** 10.18632/oncotarget.8170

**Published:** 2016-03-18

**Authors:** Chun-I Wang, Yan-Yu Chen, Chih-Liang Wang, Jau-Song Yu, Yu-Sun Chang, Chia-Jung Yu

**Affiliations:** ^1^ Molecular Medicine Research Center, Chang Gung University, Tao-Yuan, Taiwan; ^2^ Graduate Institute of Biomedical Sciences, College of Medicine, Chang Gung University, Tao-Yuan, Taiwan; ^3^ Department of Cell and Molecular Biology, College of Medicine, Chang Gung University, Tao-Yuan, Taiwan; ^4^ Department of Thoracic Medicine, Chang Gung Memorial Hospital, Linkou, Tao-Yuan, Taiwan

**Keywords:** KPNA2, lung cancer, EGFR, mTOR, E2F1

## Abstract

Karyopherin subunit alpha-2 (KPNA2) is overexpressed in various human cancers and is associated with cancer invasiveness and poor prognosis in patient. Nevertheless, the regulation of KPNA2 expression in cancers remains unclear. We herein applied epidermal growth factor (EGF) and five EGF receptor (EGFR)-related kinase inhibitors to investigate the role of EGFR signaling in KPNA2 expression in non-small cell lung cancer (NSCLC) cells. We found that EGFR signaling, particularly the mammalian target of rapamycin (mTOR) activity was positively correlated with KPNA2 protein levels in NSCLC cells. The mTOR inhibitors and mTOR knockdown reduced the protein and mRNA levels of KPNA2 in NSCLC and breast cancer cells. Specifically, rapamycin treatment induced proteasome-mediated KPNA2 protein decay and attenuated the transcriptional activation of KPNA2 by decreasing Dp1/E2F1 level *in vivo.* Immunoprecipitation assay further revealed that KPNA2 physically associated with the phospho-mTOR/mTOR and this association was abolished by rapamycin treatment. Collectively, our results show for the first time that KPNA2 is transcriptionally and post-translationally regulated by the mTOR pathway and provide new insights into targeted therapy for NSCLC.

## INTRODUCTION

The transportation of proteins and RNA into (import) and out of (export) the nucleus occurs through the nuclear pore complex and is a vital process in eukaryotic cells. Karyopherin, an evolutionarily conserved family of transport factors, mediates the nucleocytoplasmic shuttling of the large complex (>40 kDa) in cells [1]. Karyopherin subunit alpha-2 (KPNA2), a member of the karyopherin family, delivers numerous cargo proteins to the nucleus and is followed by translocation back into the cytoplasmic compartments in a Ran-GTP-dependent manner [2]. Aberrant KPNA2 expression has been observed in various human cancers, including non-small cell lung cancer (NSCLC), breast cancer, melanoma, cervical cancer, esophageal cancer, ovarian cancer, prostate cancer, liver cancer, bladder cancer, brain cancer, gastric cancer and upper tract urothelial carcinoma [3-16]. KPNA2 overexpression positively correlates with the poor prognosis of cancer patients and is associated with tumor invasiveness [8-11, 17, 18]. Although it is well-documented that KPNA2 is involved in tumorigenesis, the upstream signaling and/or transcriptional regulation of KPNA2 expression in cancer remain unclear.

Epidermal growth factor receptor (EGFR) is best known for its classical function as a receptor tyrosine kinase that is localized on the plasma membrane and activated upon ligand binding [19-22]. The activated EGFR triggers a cascade of downstream signaling molecules such as the activation of PLCγ/PKC, Ras/Raf/MEK, PI3K/Akt/mTOR and JAK2/STAT3 [22]. EGFR signaling pathways have important roles in the development of malignancy through the modulation of cell cycle progression, inhibition of apoptosis, induction of angiogenesis and promotion of tumor cell motility and metastasis [22-25]. Notably, EGFR (mutation or overexpression) and the PI3K/Akt/mTOR pathway have emerged as critical oncogenic factors in NSCLC development and progression [26].

To examine the essential role of KPNA2 protein complexes in cancer progression, we previously applied a quantitative proteomic strategy combined with immunoprecipitation to investigate the differential KPNA2 protein complexes in NSCLC cell lines with different invasiveness potential. We demonstrated that the KPNA2-vimentin-pErk complex is associated with invasiveness and that this functional complex may be regulated by the EGFR-mediated signaling pathway [27]. We proposed that the EGFR signaling pathway would be a vital regulator of KPNA2 expression. To test this hypothesis, we herein examined the effects of EGF and five different EGFR-related kinase inhibitors on the transcriptional or post-translational regulation of KPNA2 expression in NSCLC cells. We found that the suppression of mTOR significantly reduced the protein and mRNA levels of KPNA2. Importantly, we show for the first time that the mTOR pathway is involved in the regulation of KPNA2 protein turnover and correlates with the Dp1/E2F1-mediated transcription of KPNA2 in NSCLC cells.

## RESULTS

### EGFR signaling upregulates KPNA2 expression in NSCLC cells

**Figure 1:**
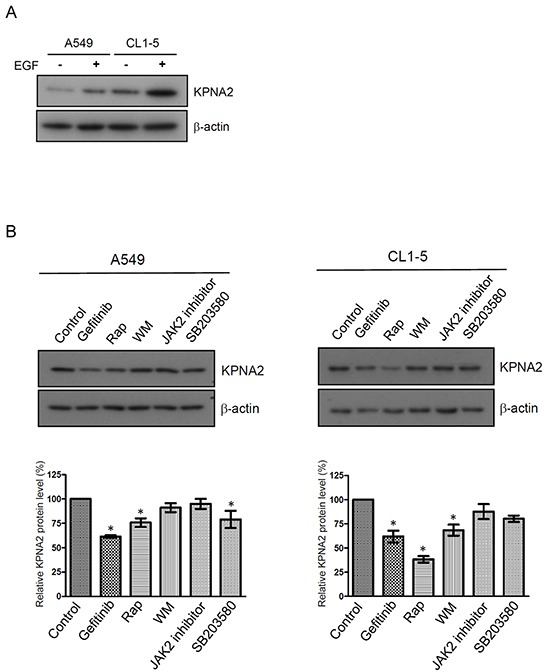
The effect of EGF and kinase inhibitor treatment on KPNA2 protein levels in NSCLC NSCLC cells were treated with/without 25 ng/mL EGF **A.** or treated with DMSO, 10 μM gefitinib, 0.5 nM rapamycin (Rap), 10 μM wortmannin (WM), 10 μM JAK2 inhibitor and 10 μM SB203580, as indicated **B.** After a 24-h treatment, cell lysates were prepared and subjected to Western blot using anti-KPNA2 and anti-β-actin antibodies. The protein signals of KPNA2 were acquired and quantified by densitometry. The signal intensities were normalized to β-actin of these proteins and presented in a histogram. The data are representative of three independent experiments. A *p* value of less than 0.05 indicates significance using the one-way ANOVA followed by Dunnett's multiple comparison test.

To investigate whether EGFR signaling mediates KPNA2 expression, we treated NSCLC cells with EGF or EGFR-related kinase inhibitors and determined KPNA2 protein levels by Western blot. Figure 1A showed that the protein levels of KPNA2 were increased upon EGF treatment in two NSCLC cell lines, A549 and CL1-5. Consistently, an EGFR tyrosine kinase inhibitor (gefitinib) or an mTOR kinase inhibitor (rapamycin) but not a JAK2 inhibitor significantly reduced KPNA2 protein levels in these two cell lines (Figure 1B). KPNA2 levels were also decreased in p38 kinase inhibitor (SB203580)-treated A549 cells and PI3K inhibitor (wortmannin)-treated CL1-5 cells. These results suggest that EGFR signaling through the mTOR pathway positively regulated KPNA2 protein levels in NSCLC cells.

### Suppression of mTOR activity reduces the mRNA and protein levels of KPNA2 in NSCLC cells

**Figure 2:**
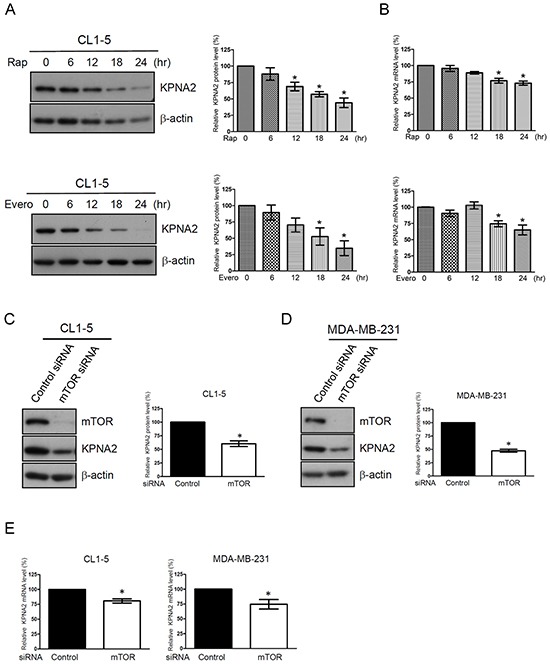
The mTOR pathway is involved in KPNA2 expression in NSCLC and breast cancer cells **A.** CL1-5 cells were treated with 0.5 nM rapamycin (Rap, upper panel) or 5 nM everolimus (Evero, lower panel) for the indicated times. After treatment, the cells were lysed and analyzed using KPNA2 antibodies by Western blot. β-actin was used as an internal control. **B.** Simultaneously, the total RNA from control or treated cells was purified and reverse-transcribed, and the resulting cDNA was subjected to qPCR analysis using Kpna2-specific primers. The mRNA level of KPNA2 was calculated as a ratio relative to control cells. **C.** CL1-5 and **D.** MDA-MB-231 cells were transfected with control and mTOR siRNA, respectively. After transfection for 72 h, cell lysates were prepared and analyzed via Western blot. β-actin was used as an internal control. **E.** Total RNA from control siRNA or mTOR siRNA-transfected cells was purified and reverse-transcribed, and the resulting cDNA was subjected to qPCR analysis using Kpna2-specific primers. The fold changes of the mRNA level of KPNA2 in mTOR-knockdown cells were calculated as a ratio relative to control siRNA-treated cells. Quantitative representation of the results obtained from three independent Western blot or qPCR analyses. A *p* value of less than 0.05 indicates significance using the one-way ANOVA followed by Dunnett's multiple comparison test (A-B) or Mann-Whitney test (C-E).

To further confirm that the mTOR pathway is involved in the regulation of KPNA2 expression, a time course experiment of rapamycin treatment and gene knockdown of mTOR were performed. Figure 2A shows that KPNA2 protein levels were significantly decreased upon rapamycin treatment for 12, 18 and 24 h. Furthermore, an approximately 25% decrease in KPNA2 mRNA levels was detected in CL1-5 cells after rapamycin treatment for 18 or 24 h (Figure 2B). We also confirmed this result by using an additional mTOR inhibitor, everolimus, to examine the suppressive effect of mTOR inhibitor on KPNA2 expression. Consistently, we found that everolimus treatment reduced the KPNA2 protein levels in a time-dependent manner (Figure 2A, lower panel), and the KPNA2 mRNA levels were decreased to 75% and 65% of control cells upon everolimus treatments for 18 and 24 h, respectively (Figure 2B, lower panel). Furthermore, mTOR knockdown significantly reduced the protein and mRNA levels of KPNA2 in CL1-5 cells (Figure 2C and 2E). To examine whether this event was specific to lung cancer cells, we performed the same experiments using a breast cancer cell line, MDA-MB-231. As shown in Figure 2D and 2E, mTOR knockdown also reduced the protein and mRNA levels of KPNA2 in MDA-MB-231 cells. These results suggest that the mTOR activity was positively correlated with KPNA2 gene and protein expressions and that this characteristic was not specific to lung cancer cells.

### Rapamycin treatment increases KPNA2 turnover in NSCLC cells

**Figure 3:**
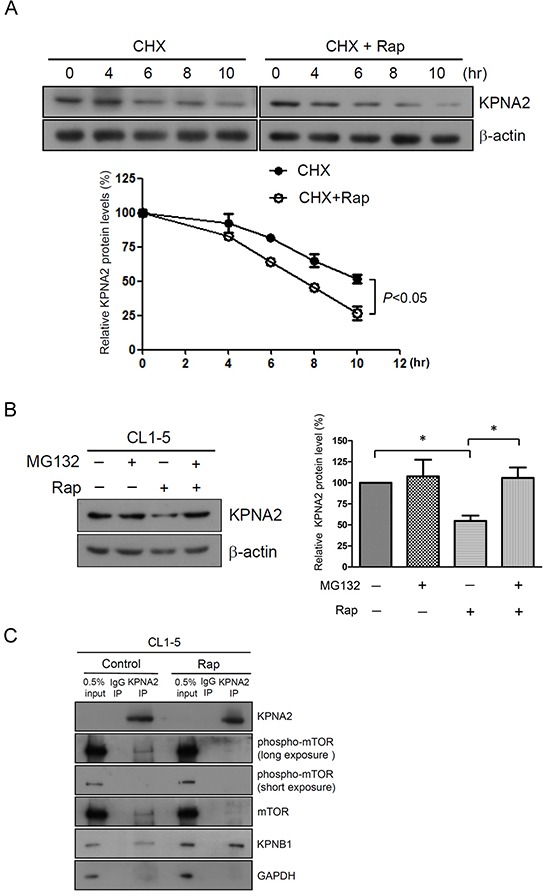
Rapamycin treatment increases KPNA2 turnover in NSCLC cells **A.** Analysis of the half-life of KPNA2 protein levels in 100 μg/ml cycloheximide (CHX)-treated CL1-5 cells with or without 0.5 nM rapamycin (Rap) for the indicated times. Following treatment, the cells were lysed and analyzed using KPNA2 antibodies by Western blot. Quantitative representation of the results obtained from three independent experiments. The error bars indicate the SEM. A *p* value of less than 0.05 indicates significance using a two-way ANOVA test. **B.** CL1-5 cells were treated with 0.5 nM Rap in the absence or presence of 1 μM MG132 for 18 h. After treatment, the cells were lysed and detected using KPNA2 antibodies by Western blot. β-actin was used as an internal control. Quantitative representation of the results obtained from three independent experiments. A *p* value of less than 0.05 indicates significance using the one-way ANOVA followed by Tukey's post-hoc test. **C.** mTOR and phospho-mTOR were associated with KPNA2 *in vivo*. CL1-5 cells were treated with or without Rap (10 nM) for 15 min followed by co-immunoprecipitated using anti-KPNA2 antibody, as described in the Materials and Methods. The precipitated protein complexes were analyzed by Western blot using antibodies against phospho-mTOR, mTOR, KPNB1 and KPNA2, as indicated. KPNB1 was used as a positive control of a KPNA2-interacting protein. GAPDH was used as an internal control.

Interestingly, the protein, but not the mRNA levels of KPNA2 were significantly decreased in NSCLC cells upon rapamycin treatment for 12 h (Figure 2A and 2B). We next examined whether mTOR induced KPNA2 protein decay by determining changes of KPNA2 levels in cells that were treated with cycloheximide. The half-life of KPNA2 in the presence of cycloheximide was approximately 10 h, whereas the half-life of KPNA2 was reduced to approximately 8 h when cells were co-treated with cycloheximide and rapamycin (Figure 3A). In addition, the rapamycin-induced KPNA2 decrease was abolished in the presence of the proteasome inhibitor MG132 (Figure 3B), suggesting that the mTOR pathway modulated the proteasome-mediated KPNA2 degradation in NSCLC cells. Notably, previous studies have shown that KPNA1 (a STAT1 karyopherin) interacts with mTORC1 in a complex that includes STAT1 and the mTOR-associated phosphatase PP2Ac [29]. To investigate whether mTOR modulated KPNA2 degradation through a physical association with KPNA2, we determined the association between KPNA2 and phospho-mTOR/mTOR by an immunoprecipitation assay. As shown in Figure 3C, phospho-mTOR/mTOR was detected in the KPNA2-immunoprecipitated complex in CL1-5 cells; however, this association was abolished by rapamycin treatment. These results indicate that mTOR associated with KPNA2 in a macromolecular complex, which might result in the modulation of proteasome-mediated KPNA2 degradation.

### Rapamycin treatment attenuates the transcriptional activation of KPNA2 by decreasing Dp1/E2F1 level in NSCLC cells

**Figure 4:**
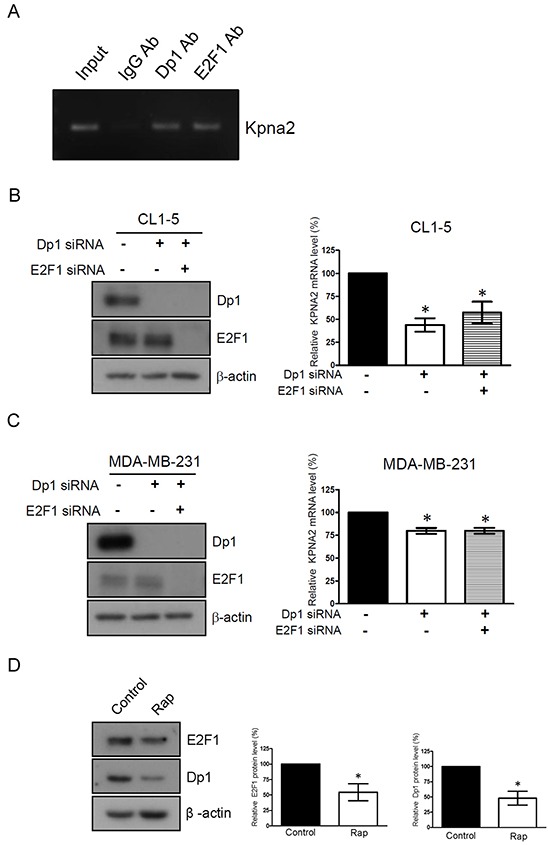
Rapamycin treatment attenuates the transcriptional activation of KPNA2 by decreasing Dp1/E2F1 level in NSCLC cells **A.** Fragmented chromatins prepared from CL1-5 cells were immunoprecipitated with IgG, Dp1 or E2F1 antibodies, as indicated. DNA isolated from immunoprecipitated material was amplified by PCR using primers that spanned the Dp1 and E2F1 sites, which were present in the Kpna2 promoter. Amplified fragments were analyzed by electrophoresis on a 2% agarose gel. **B.** CL1-5 and **C.** MDA-MB-231 cells were transfected with control, Dp1 and E2F1 siRNA, as indicated. After transfection for 48 h, cell lysates were prepared and analyzed using Western blots. Simultaneously, total RNA from control siRNA, Dp1 or Dp1/E2F1 siRNA-transfected cells was purified and subjected to qPCR analysis using KPNA2 gene-specific primers. Fold change of the mRNA level of KPNA2 in Dp1- or Dp1/E2F1-knockdown cells was calculated as a ratio relative to control siRNA-treated cells. **D.** CL1-5 cells were treated with 0.5 nM rapamycin (Rap) for 24 h. After treatment, the cells were lysed and analyzed using E2F1 and Dp1 antibodies by Western blot. β-actin was used as an internal control. Quantitative representation of the results obtained from three independent of qPCR or Western blot analyses. A *p* value of less than 0.05 indicates significance using one-way ANOVA followed by Dunnett's multiple comparison test (B-C) or Mann-Whitney test (D).

Little is known regarding the transcriptional regulation of KPNA2 in cancer cells. Recently, van der Watt *et al.* reported that the Kpna2 promoter contains functional E2F sites and that E2F/Dp1 heterodimers bind and activate the Kpna2 promoter in cervical cancer cells [30]. To determine whether E2F/Dp1 heterodimers bind and activate the Kpna2 promoters in the NSCLC cell line, ChIP assays were performed using chromatin prepared from CL1-5 cells. DNA was immunoprecipitated using Dp1 or E2F1 antibodies and amplified by PCR with primers that spanned the Dp1 or E2F sites in the Kpna2 promoters, respectively. Positive amplification in the CL1-5 cell line confirmed an association between Dp1 and E2F1 sites in the Kpna2 promoter (Figure 4A). Furthermore, to determine whether the binding of E2F1/Dp1 to the Kpna2 promoter activated Kpna2 transcription *in vivo*, E2F1 activity was inhibited by silencing the expression of Dp1 alone or by co-silencing with E2F1 in CL1-5 cells using the siRNA gene knockdown approach. Western blot analysis showed that Dp1 and E2F1 protein levels were dramatically decreased in Dp1- and Dp1/E2F1-knockdown cells. Simultaneously, we found that the mRNA levels of KPNA2 were significantly decreased in Dp1- and Dp1/E2F1-knockdown cells (Figure 4B). Interestingly, this event was also observed in MDA-MB-231 cells (Figure 4C), suggesting that E2F/Dp1 heterodimers bind and activate the Kpna2 promoter in several types of cancer cells, including NSCLC, breast and cervical cancer cells. To further elucidate whether rapamycin attenuated the transcriptional activity of KPNA2 through suppressing the expressions of Dp1 or E2F1, we treated CL1-5 cells with rapamycin and examined the effects on Dp1 and E2F1 expressions. As shown in Figure 4D, rapamycin treatment significantly reduced the protein levels of Dp1 and E2F1 in NSCLC cells. This result indicates that rapamycin treatment suppressed the transcriptional activity of KPNA2 via the down-regulation of Dp1 and E2F1 expression.

## DISCUSSION

KPNA2 has been implicated in the translocation of a variety of proteins that are associated with tumor-suppressive as well as oncogenic properties, including p53, c-Myc, E2F1, Oct4, and p65 [31-35]. It is reasonable to suggest that KPNA2 overexpression in cancer cells may perturb a variety of processes through the altered shuttling of its nuclear cargo proteins, thereby supporting carcinogenesis. Accordingly, elucidating the underlying mechanism of the regulation of KPNA2 expression is critical for understanding lung cancer progression. In the current study, we show that mTOR activity was positively correlated with KPNA2 expression. Specifically, rapamycin treatment significantly reduced KPNA2 expression through the acceleration of KPNA2 protein turnover and the suppression of Dp1/E2F1 expression in NSCLC cells. However, the protein levels of KPNA2 only decreased by approximately 25% upon rapamycin treatment in A549 cells (Figure 1B), and KPNA2 mRNA decreased by 20% (Figure 2B). This result suggests that signaling beyond the mTOR pathway may also contribute to KPNA2 expression. Nevertheless, our results collectively demonstrate that the mTOR signaling pathway has a central role in regulating the transcriptional and post-translational expression of KPNA2 in NSCLC cells.

It has been reported that seventeen components of the mTOR pathway, including seven oncoproteins (RAF, MAPK, CTNNB1, PI3K, AKT, MDM2 and CDK) and ten tumor suppressor proteins (NF1, GSK3, APC, TSC1/2, PTEN, ATM, STK11, TP53, CDKN2A and RB1) are mutated in more than 30% of lung adenocarcinoma sequenced, not including tumors with KRAS mutations [36]. Notably, activation of the PI3K/AKT/mTOR pathway in NSCLC results in a more aggressive disease that correlates with poor prognosis for patients and causes resistance to agents that target upstream receptor tyrosine kinases [37, 38]. This result suggests that mTOR is important for lung carcinogenesis and is a potential therapeutic target. Therefore, rapamycin and several of its derivatives that target the mTOR pathway, such as temsirolimus (CCI-77), everolimus (RAD001) and ridaforolimus (AP23573), have been developed as potential targeted therapeutic agents in NSCLC [37, 39-44]. In the current study, we found that rapamycin induced proteasome-mediated KPNA2 degradation (Figure 3A-3B). Phospho-mTOR/mTOR was also detected in the KPNA2-immunoprecipitated complex (Figure 3C), which indicated that the induction of KPNA2 protein decay is a possible mechanism of rapamycin functioning in anti-cancer processing. Considering that KPNA2 is overexpressed in lung adenocarcinoma and squamous cell carcinoma, which is the most common type of NSCLC that involves a deregulated PI3K/AKT/mTOR pathway [6, 45-48], we proposed that KPNA2 would be a potentially predictive biomarker of responsiveness to PI3K/AKT/mTOR-targeted therapy in NSCLC. Furthermore, this possibility is worthy of further investigation.

Our previous study indicated that KPNA2 interacts directly with E2F1 and mediates E2F1 transport from the cytoplasm to the nucleus [31]. We herein demonstrated that Kpna2 promoter contains Dp1/E2F1-binding sites in NSCLC cells (Figure 4). These findings raise the possibility that a positive feedback loop exists, where the elevated activity of E2Fs may result in an increased expression of KPNA2, thereby increasing the nuclear amounts of E2Fs. Both KPNA2 and E2F1 are overexpressed in lung cancer and other human cancers, and the study presented here highlights that this positive feedback loop may provide a new avenue for cancer targeted therapy.

## MATERIALS AND METHODS

### Cell culture

The human lung adenocarcinoma cancer cell line CL1-5 was kindly provided by Professor P.C. Yang (Department of Internal Medicine, National Taiwan University Hospital, Taipei, Taiwan, R.O.C.) [28]. CL1-5 cells were maintained in RPMI 1640 (Gibco, Invitrogen, Carlsbad, CA, USA). A549 and MDA-MB-231 cells were maintained in DMEM (Gibco). All media contained 10% FBS plus antibiotics, and the cells were maintained at 37°C in a humidified atmosphere of 95% air/5% CO_2_.

### Reagents and antibodies

Epidermal growth factor (EGF), rapamycin, wortmannin, JAK2 inhibitor and SB203580 were purchased from Millipore (Bedford, MA, USA), and gefitinib was purchased from Tocris (Ellisville, MO, USA). Everolimus and cycloheximide were purchased from Abcam (Cambridge, MA, USA) and Sigma (St. Louis, MO, USA), respectively. These compounds were dissolved in dimethyl sulfoxide (DMSO) and diluted in fresh media before each experiment. The final DMSO concentration was less than 0.1%. The antibodies used in this study and their sources were as follows: the KPNA2 antibody (B-9), KPNB1 antibody (H-300), Dp1 antibody (K-20) and E2F1 antibody (KH95) were purchased from Santa Cruz Biotechnologies (Santa Cruz, CA); the mTOR and phospho-mTOR (S2448) antibodies were purchased from Cell Signaling (Beverly, MA, USA); and the β-actin antibody was obtained from Millipore (Bedford, MA, USA).

### Gene knockdown of mTOR, Dp1 and E2F1 using small interfering RNA

Gene knockdown of mTOR, Dp1 or E2F1 was performed using small interfering RNA (siRNA). Briefly, 19-nucleotide RNA duplexes that targeted human mTOR or E2F1 were synthesized and annealed by Dharmacon (Thermo Fisher Scientific, Lafayette, CO). The 21-nucleotide RNA duplexes that were used to target human Dp1 were purchased from Santa Cruz. Briefly, CL1-5 or MDA-MB-231 cells were transfected with control siRNA or mTOR-pooled siRNA (GGCCAUAGCUAGCCUCAUA, CA AGGACUUCGCCCAUAA, GCAGAAUU GUCAAG GGAUA, CCAAAGCACUACACUACAA), E2F1-pooled siRNA (UCGGAGAACUUUCAG AUCU,GAGAAGUC ACG CUAUGAGA, GAGCAGAUGGUUAUGGUGA, GAACAGGGCCACUGACUCU) or Dp1-pooled siRNA (CCACUUCCUACA ACGAAGUTT, CGAUGACUUCA ACGAGAAUTT, GCAUCUUCCUGUAAUCUAUTT) using Lipofectamine RNAiMAX reagents (Invitrogen) according to the protocol provided by the manufacturer. At 48 h after transfection, the cell lysates were prepared for Western blot to determine the gene knockdown efficacy.

### Immunoprecipitation assay

CL1-5 and MDA-MB-231 cells were extracted in Nonidet P-40 (NP-40) lysis buffer [1% NP-40, 20 mM Tris-HCl (pH 7.5), 150 mM NaCl, 1 mM Na_3_VO_4_, 5 mM EDTA (pH 8.0), 10% glycerol, 10 μg/mL leupeptin, 10 μg/mL aprotinin, 1 mM PMSF] and fractionated by centrifugation (13,000 rpm, 10 min at 4°C) to obtain cell lysates. For immunoprecipitation of the endogenous KPNA2, cell lysates (2 mg of protein) from CL1-5 and MDA-MB-231 cells were incubated in 4 μg of an anti-KPNA2 antibody (B-9; Santa Cruz Biotechnology) or control IgG (Santa Cruz Biotechnologies) together with 20 μL of Dynabeads protein G (Invitrogen). All incubations were performed for 2 h at room temperature with rotation, and the samples were then washed twice with Tris buffer A [20 mM Tris-HCl (pH 7.5), 250 mM NaCl, and 0.5 mM DTT] and three times with Tris buffer B [20 mM Tris-HCl (pH 7.5) and 0.5 mM DTT]. The resulting protein complexes were eluted with SDS sample buffer, separated via SDS-PAGE, transferred to PVDF membranes, and analyzed by Western blot using primary antibodies against the following proteins: phospho-mTOR (S2448), mTOR, KPNB1 and KPNA2. Subsequently, the membranes were incubated with the appropriate secondary antibodies, and the signals were visualized by enhanced chemiluminescence according to the manufacturer's specifications (Millipore Inc., Billerica, MA, USA).

### Chromatin immunoprecipitation (ChIP) assay

Cells were grown to approximately 90% confluency, and protein-DNA complexes were cross-linked with 1% formaldehyde for 10 min followed by the addition of 0.125 M glycine for 10 min. The cells were harvested, lysed in lysis buffer [1% NP-40, 0.1% SDS, 5 mM EDTA, 150 mM NaCl, 0.5% deoxycholate, 50 mM Tris, pH 8.0, 2% cOmplete Protease Inhibitor Cocktails (Roche)], and sonicated to lengths between 400 and 1000 bp. The crosslinked, sonicated chromatin was precleared with 10 μL of Dynabeads protein G (Invitrogen) before incubation with 2 μg of the indicated antibodies and rotated at 4°C overnight. Dynabeads protein G were added for an additional 15 min. Normal mouse IgG antibody (Santa Cruz Biotechnology) was used as the control immunoprecipitation. After extensive washes, immunocomplexes were treated with Proteinase K and decrosslinked at 65°C for 6 h. Bound DNA in the ChIP was extracted by the PCR purification kit (Qiagen, Chatsworth, CA, USA) and subjected to PCR analysis using primers that were designed to span the Dp1 and E2F1 binding sites (Kpna2 F: ATGGGCACACAGCTTAG; Kpna2 R: CTGAGTCTGTACCTGCGAA). After amplification, PCR products were separated on a 2% agarose gel and were analyzed under UV light to view the stained DNA.

### Statistical analyses

All data were processed using SPSS 12.0 (SPSS Inc., Chicago, IL, USA). All continuous variables were expressed as the mean ± standard deviation (SD) or standard deviation of the mean (SEM). For quantitative analysis of the protein decay, two-way ANOVA was used. The Mann-Whitney test or one-way ANOVA was used to analyze the quantification results obtained from Western blot and qPCR analyses.

## Abbreviations

KPNA2: karyopherin subunit alpha-2
EGFR: epidermal growth factor receptor
NSCLC: non-small cell lung cancer
EGF: epidermal growth factor
ChIP: chromatin immunoprecipitation
mTOR: mammalian target of rapamycin
qPCR: real-time quantitative PCR
DMSO: dimethyl sulfoxide
KPNB1: karyopherin subunit beta-1
